# Genotypic and environmental effect on male flower production in *Cupressus sempervirens* clones and selection of genotypes with reduced pollen emission

**DOI:** 10.3389/fpls.2022.1032200

**Published:** 2022-11-01

**Authors:** Roberto Danti, Sara Barberini, Vincenzo Di Lonardo, Gianni Della Rocca

**Affiliations:** Istituto per la Protezione Sostenibile delle Piante, Consiglio Nazionale delle Ricerche (IPSP-CNR), Sesto Fiorentino, Italy

**Keywords:** cypress, plant breeding, heritability, pollen allergies, pollinosis, stability

## Abstract

Common cypress (*Cupressus sempervirens* L.) is widespread in the Mediterranean area and is frequently planted as ornamental tree in parks and gardens. Like other species of *Cupressus*, common cypress releases a significant amount of the total annual airborne pollen in most regions and is known as responsible for winter pollinosis. Although variation in the production and release of pollen has been observed among *C. sempervirens* trees growing in urban areas, no information is available on effects due to genotype × environment interaction on this trait. In this study more than 150 C*. sempervirens* clones were analyzed for two to four consecutive years in clonal orchards situated in central Italy to evaluate variations in the production of male cones. Variance component ANOVA underscored an important genetic control of male flowering, with high repeatability (from 0.80 to 0.95) found in single environments. Analysis for combined sites or years (in a single site) showed significant effect of environment and genotype × environment interaction on the total variance. Intra-trait genetic correlations between environments were moderate to high (from 0.40 to 0.92), which indicates that male cone production of clones is fairly consistent across years and sites. Of the 10 clones characterized by the lowest mean male cone production, three showed good stability across environments based on the linear regression coefficient and Wricke’s ecovalence. The mean cone production of these 10 clones was 5 to 10 times lower than the mean production observed in the same environment. These clones have both ornamental and hypoallergenic traits and hold promise for designing green spaces with low allergy impact.

## Introduction

Pollen released by vegetation, one of the main components of bioaerosol, is directly involved in respiratory allergies. About 30% of the world’s population is affected by respiratory diseases caused by atmospheric pollen (Brunekreef et al., 2000; Linneberg et al., 2016), with significant economic and social consequences (Ishizaki et al., 1987; Marcellusi et al., 2015). An increase in respiratory pollen allergies is expected in the Mediterranean as a result of the effects of environmental change on the geographic distribution of species and on the pattern of emission of pollen allergens (Cramer et al., 2018). In urban areas, the situation is exacerbated by the effect of heat islands and air pollution on the reproductive phenology of plants (Zipper et al., 2016) and the expression of pollen allergens (D’Amato, 2001; Bielory et al., 2012; D’Amato et al., 2014; Mattei et al., 2022). An interaction between air pollution and plant-derived respiratory disorders has also been observed (D’Amato et al., 2010; D’Amato et al., 2014).

Pollen of Cupressaceae is known for its important role as an airborne allergen in the Mediterranean. In this region, common cypress (*Cupressus sempervirens* L.) is widespread as a native or cultivated species, planted mainly for ornamental and landscaping purposes, reforestation, wood production, and as windbreaks (Andreoli and Xenopoulos, 1990; Xenopoulos et al., 1990; Della Rocca et al., 2007). This tree is widely used in parks and urban green areas in many Mediterranean countries because it has aesthetic value, has little need for water, and is inexpensive to maintain (Danti et al., 2006; Della Rocca et al., 2007).

Like most Cupressaceae species, *C. sempervirens* is a monoecious tree, bearing both reproductive organs (male and female) separately on the same tree at the ends of short branchlets (Farjon, 2005). In *C. sempervirens* the first flowering occurs early, starting at 3–4 years of age and even earlier on grafts. Male (pollen) cones are terminal on ultimate branchlets and are made of 8–16 decussate microsporophylls, each bearing 3–6 microsporangia that contain pollen grains (Farjon, 2005).

Being an anemophilous species, common cypress releases a huge amount of pollen that is dispersed by wind and that has been recognized as responsible for a significant amount of the total annual airborne pollen in the Mediterranean (D’Amato et al., 2007). In this region, *Cupressus* and *Olea* produce the largest amount of allergenic tree pollen, and *Cupressus* pollen accounts for 14% to 40% of the total annual pollen count (Charpin et al., 2019). Cypress pollen contains the allergens responsible for winter pollinosis, which is marked by an onset of inflammation affecting the upper respiratory tract during January–April and increased susceptibility to respiratory allergies. The prevalence of cypress pollinosis ranges from 0.6% to 3% in the general population (Charpin et al., 2019). In central Italy, cypress pollen is the main sensitizing allergen, and 63% of patients are allergic to cypress pollen (Sposato et al., 2014). This high incidence of pollinosis is due to the proximity of cypress to dwellings and to exposure to increasing amounts of pollen coupled with higher expression of pollen allergens due to air pollution in urban sites (Suárez-Cervera et al., 2008; D’Amato et al., 2010; Sénéchal et al., 2015; Mattei et al., 2022).

Pollen production is generally influenced by biotic and abiotic factors (La Deau and Clark, 2006; Rogers et al., 2006). With regard to the latter, meteorological conditions, and chiefly air temperature, have significant effects at both the macro- and micro-climatic scales (Faegri and Iversen, 1989; Moore et al., 1991; Damialis et al., 2011). Aerobiological investigations have been conducted over the past decade to monitor the presence of Cupressaceae allergenic pollen in urban areas. In particular, some studies have focused on the prediction and modeling of local pollen production based on climate data and the charting of the phenology of male flowers of *Cupressus* species (Hidalgo et al., 2003; Torrigiani-Malaspina et al., 2007; Dalla Marta et al., 2011). In conifers pollen production can also be influenced by tree size and age, branching habit (Leslie, 2012), pruning (Stoehr et al., 1995), annual cycles of fecundity, and even interactions with pollen-feeding insects (Caron and Powel, 1989; McCullough, 2000; Ne’eman et al., 2010).

Studies that have monitored the flowering of cypress plants near urban areas have reported marked differences in male cone production among and within species (Hidalgo et al., 1999; Aboulaich et al., 2008) but showed a relatively stable number of pollen grains per cone within a single species and among *Cupressus* species (Hidalgo et al., 1999; Damialis et al., 2011). Hidalgo et al. (1999) suggested that in *Cupressus* species the distribution of flowers around the crown depends on both environmental and genetic factors. Several studies have highlighted the role of both genotype and environment in the production of both male and female flowers in different conifer species in clonal orchards (Saito and Kawasaki, 1984; Burczyk and Chalupka, 1997; Kang, 2000; Nikkanen and Ruotsalainen, 2000; Choi et al., 2004; Bilir et al., 2006).

Despite the fact that certain variation in the production of male cones has been detected among mature *C. sempervirens* trees growing in urban areas (Aboulaich et al., 2008; Damialis et al., 2011), no information is available on effects due to genotype or environment and genotype × environment (G×E) interaction.

G×E interaction, defined as the differential response of genotypes in different environments (Falconer, 1989), complicates testing and selection in breeding programs. However, the simple evaluation of this variance component does not provide detailed indications of the behavior of genotypes in different environments. Assessing the stability and adaptability of genotypes/clones across environments can assist selection. A cypress breeding program conducted in Italy by IPSP-CNR has been mainly based on the evaluation of clones in experimental plots. The use of clones, obtained *via* vegetative propagation (grafting), allows the replication of selected traits, such as resistance to cypress canker disease, habitus, and growth, which are important for ornamental purposes, for the production of wood, and for windbreaks (Raddi and Panconesi, 1991; Danti et al., 2006; Danti et al., 2013; Nocetti et al., 2015; Nocetti et al., 2017). The use of clones allows for more accurate evaluation of G×E interaction and genotypic stability compared to the use of seed progenies (Bentzer et al., 1988).

The overall objective of this study was to assess the male cone production of 20-year-old *C. sempervirens* var. *stricta* clones (with a fastigiated habit) growing in two plots established in two sites in central Italy (Tuscany and Umbria) and suited for ornamental planting and landscaping. Clones were assayed over consecutive flowering seasons to 1) investigate effects due to genotype and environment and their interaction on male cone production, 2) evaluate the stability of clones, and 3) possibly select stable clones showing null or low pollen release. The selection of plant genotypes that have both ornamental and hypoallergenic traits may be very useful for designing green spaces with low allergy impact.

## Material and methods

The plant material used herein consisted of 153 20-year-old *C. sempervirens* (var. *stricta*) clones that had been previously planted to assess their response to bark canker (Danti et al., 2006; Danti et al., 2013; Danti and Della Rocca, 2017). The trial was set up in two experimental plots in central Italy with different soil and climate conditions. Plants growing in the same plot were very similar in terms of trunk height and diameter. Clones whose plants showed abnormal growth were excluded from the observations. The first plot was located at Roselle (province of Grosseto, Tuscany; 42°48′ N, 11°05′ E; 5 m asl) on sandy loam, well-drained soil. It was characterized by a typical Mediterranean climate with long, dry summers and relatively mild winters. From 1996 to 2016 (20 years) the average rainfall was 650.2 mm/year distributed over 69 rainy days, with a peak in autumn (November); the yearly average temperature was 15.0°C (according to the archives of the Hydrographic Service of Regione Toscana). The second plot was located in Cannara (province of Perugia, Umbria; 43°00′ N, 12°37′ E; 188 m asl) on reclaimed but clayey soil in the nursery UmbraFlor s.r.l. It was characterized by a moderately continental climate with hot summers and cold winters with sporadic snowfall. In the same 20-year period (1996–2016), the average rainfall was 874 mm/year distributed over 83 rainy days, with peaks in autumn and spring (November and April); the yearly average temperature was 13.8°C (archives of the Hydrographic Service of Regione Umbria). The experimental fields in both sites were completely cleared and cultivated. Temperature and rainfall data recorded for both sites from 2012 to the end of the surveys are reported in **Supplementary Table 1**.

In each plot, common cypress (*C. sempervirens* L.) clones were planted in 1995 at 3 × 3 m spacing, and no thinning occurred until the time of the survey. For each clone, ramets were obtained through scions collected in January 1993 from vigorous and healthy *C. sempervirens* var *stricta* ortets selected in ornamental and landscape plantations in central Italy regardless of their production of male cones and pollen. Scions were immediately grafted on 1-year-old *C. sempervirens* seed rootstocks grown in small pots containing a mixture of 3 peat/1 compost/1 perlite (v/v/v). Grafts were kept in the greenhouse until the end of spring, when they were transplanted into larger pots (4–5 L) and kept for 2 years in a shading tunnel before being transplanted in the field (Danti et al., 2013).

### Estimation of male cone production

In Cannara, surveys were conducted over four consecutive years, from 2013 to 2016, on three to five ramets for each of 98 clones. Surveys were repeated for 4 years to obtain a reliable representation of seasonal fluctuations in male cone production. In Roselle, from three to nine ramets for each of 153 clones were examined in 2013 and 2014 (in 2015 the surveys had to be stopped, as the plot was dismantled because of a change in ownership). All clones surveyed in Cannara were also present in Roselle. A total of 1832 trees were assessed for male cone production.

To estimate male cone production, each tree was surveyed two times per week during the flowering season (from the end of January to the beginning of April). In particular, the phases of full flowering and the start of pollen emission were characterized for each tree examined based on the phenophases chart proposed by Hidalgo et al. (2003).

The production of male cones was evaluated for each tree when near to flowering and in full flowering, phenophases 3 and 4, respectively, based on Hidalgo et al. (2003). Trees were first rated based on visual assessment of the percentage of the crown covered by male cones, i.e., the number of mature male cones, produced on the total volume of the tree subdivided into eight flower density categories. For each category, three trees were sampled to quantify the number of male cones per branch. Because the distribution of male cones generally varied at different heights within the same crown and among twigs, sampling was carried out by subdividing the crown into three heights (lower, medium, and upper). For each of the three heights, three random twigs falling inside a 0.5 m^2^ square frame laid down on the crown surface in each of the four cardinal directions were sampled (12 samples per tree). For each twig sampled (20–30 cm long on average) the number of male cones was counted and the number of branchlets (tips) that could bear a male cone was estimated. For the eight categories of tree the mean ratio between the number of cones and the number of branchlets measured on the 36 twigs collected from the three crown heights and the four crown directions was defined as follows:

1: male cones absent or very few (rare) and isolated, not detectable by the naked eye (at a distance) and practically with little contribution to pollen load;
2: ≤ 1%, very low producing on scattered twigs;
3: 2–5%, low male cone producing: male cones sparse and scattered and not evenly distributed throughout the crown;
4: 6–10%, moderate cone producing;
5: 11–15%, medium cone producing;
6: 16–20%, high cone producing: more or less dense on significant portions of the crown;
7: 21–25%, very high cone producing on most portions of the crown; and
8: 26–40%, very high cone producing on most portions of the crown.

### Count of pollen grains per male cone

Three individual male cones were harvested immediately before opening from three different branches of three ramets of clones that had previously been classified as low producing cones (categories 1 or 2) or high producing cones (categories 6, 7, or 8). They were brought to the laboratory inside small paper bags and were processed within 24 h of harvesting. Male cones were then crushed in an Eppendorf tube, hydrated with 1 mL water, vortexed, and immediately counted under a light microscope (100X, Zeiss Axioscop 50) using a Burker hemocytometer (Godini, 1981). Before being crushed, male cones were measured with a Stereoscope (10X, Nikon SMZ800) equipped with a digital camera.

### Statistical analysis and estimation of genetic parameters

**Figure 1:**
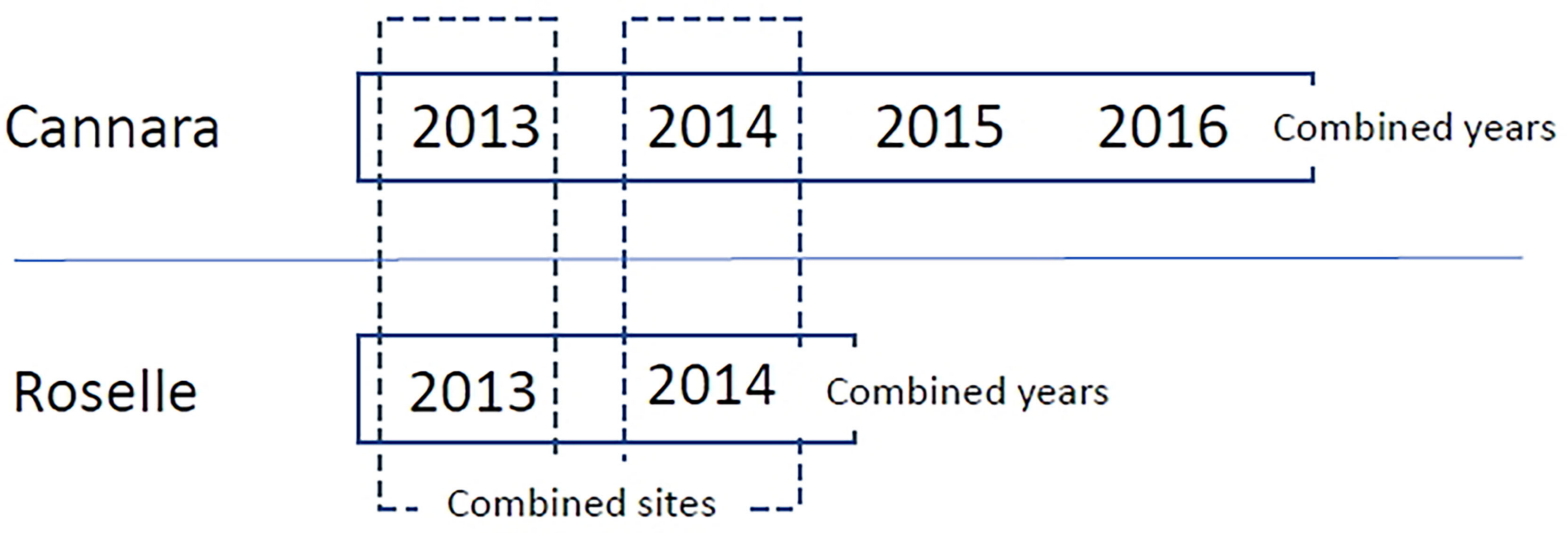
Scheme of ANOVA analysis followed for combined environments. For both sites, each year represents a single environment. So, there are 4 environments in Cannara and 2 in Roselle. From the combination of site per year we obtain a total of 6 environments. For each of the two sites, combined analyzes were carried out for years (represented by rectangles with solid lines) to study the variations of clones in different years in the same site. While between the two sites, combined analyzes were carried out in 2013 and 2014 (represented by the dashed rectangles) to study the effect of the site in two different years.

The study aimed at evaluating variations in male cone production of clones within a single site, between years in a single site, and between two sites. Scores from 1 to 8 recorded for the surveyed ramets in Roselle and Cannara were subjected to analysis of variance (ANOVA) using the General Linear Model of the Statistica 10 package. In all, six environments were considered based on combinations of year and site (4 years for Cannara and 2 years for Roselle). Data were first analyzed separately for single environments by one-way ANOVA to evaluate differences among clones in each site × year combination. Then data were analyzed for combined environments by two-way ANOVA to evaluate effects due to genotype, environment (sites or years within a single site), and G×E interaction on male cone production of common cypress clones, as reported in **Figure 1**.

Homogeneity of variance was tested with Bartlett’s test for single environment analysis and with joint analysis for site and year.

Mixed-model analysis was used to calculate variance components following the expected mean squares (MS) method (ANOVA estimates) and assuming environment (sites and years in a single site) as a fixed effect and genotype and G×E interaction as random effects.

Variance components of random effects were calculated for single environments (single years within sites) and for joint environments (the two sites combined and years combined within a single site) as in **Figure 1**. The repeatability (broad sense heritability) of clone means ( $R_{c}^{2}$ ) in a given environment was calculated using the following formula (Zhang et al., 2003; Pliura et al., 2007):


(1) $$R_{c}^{2}=\frac{\sigma_{c}^{2}}{\sigma_{c}^{2}+\frac{\sigma_{e}^{2}}{r}},$$

where *r* is the number of replications per clone.

The individual-tree clonal repeatability ( $R_{b}^{2}$ ) was estimated using the following equation:


(2) $$R_{b}^{2}=\frac{\sigma_{c}^{2}}{\sigma_{c}^{2}+\sigma_{e}^{2}}.$$

The repeatability of clone means across environments (sites or years in a single site) was calculated as the ratio of the genotypic (clonal) variance component ( $\sigma_{c}^{2}$ ) to the total phenotypic variance of all random effects, according to the following formula:


(3) $$R_{c}^{2}=\frac{\sigma_{c}^{2}}{\sigma_{c}^{2}+\frac{\sigma_{ExC}^{2}}{q}+\frac{\sigma_{e}^{2}}{rq}},$$

where $\sigma_{ExC}^{2}$ is environment-clone interaction variance, $\sigma_{e}^{2}$ is the residual variance, and *q* and *r* are the number of environments (sites or years) and the number of replications per clone per environment (site or year), respectively.

The individual-tree clonal repeatability ( $R_{b}^{2}$ ) across environments (sites or years) was estimated as


(4) $$R_{b}^{2}=\frac{\sigma_{c}^{2}}{\sigma_{c}^{2}+\sigma_{ExC}^{2}+\sigma_{e}^{2}}.$$

The standard errors for repeatability estimations were calculated with the following formula (Becker, 1984):


(5) $$SE\left(R^{2}\right)=\sqrt{\frac{2{\left(1-R^{2}\right)}^{2}{\left[1+\left(k-1\right)R^{2}\right]}^{2}}{k\left(k-1\right)\left(N-1\right)}},$$

where *R^2^* is the repeatability estimate, *k* is the coefficient associated with the variance due to clonal variation, and *N* is the number of clones tested.

Genetic correlations between clonal means in pairs of environments were calculated, with the scores recorded for a clone in two different environments (sites or years) considered two distinct traits. The following formula was used to estimate genetic correlations (type B genetic correlations; Burdon, 1977):


(6) $$r_{B}=\frac{r_{p\left(x1,\;\;x2\right)}}{\sqrt{R_{c\left(x1\right)}^{2}R_{c\left(x2\right)}^{2}}}$$

where *r*_*p*(*x*1,  *x*2)_ is the coefficient of correlation between clone mean scores measured in environments 1 (*x*_1_) and 2 (*x*_2_) and $R_{c\left(x1\right)}^{2}\;$ and $R_{c\left(x2\right)}^{2}$ are the clonal mean repeatability of the score at environments *x*_1_ and *x*_2_ (site × year combination), respectively.

### Assessment of clonal stability

The dynamic concept of stability was used in this study to evaluate how clones responded to the six environments considered. This concept assumes that a genotype is stable if its response to environmental changes does not deviate from the general response of all genotypes in the trial. Two stability parameters were calculated: the linear regression coefficient and Wricke’s ecovalence. The linear regression coefficient *b_i_* (Finlay and Wilkinson, 1963) was used following Yu and Pulkkinen (2003) to estimate the performance of individual clones against site means and evaluate their stability and adaptability based on how a genotype responds to a range of environments. We calculated the regression coefficient *b_i_* using the following regression model:


, $$y_{i}=a+bx_{i}+e_{i}$$

where *y_i_* is the clonal value at site *i*, *a* is the intercept of the clone, *x_i_* the average of all clones at the site, and *e_i_* is the unknown error. A *b_i_* value close to 1 indicates average stability across sites; *b_i_* > 1 indicates low stability, which means that the clone is responsive to favorable environments. *b_i_* < 1 indicates high stability, which means that the clone is less responsive to changing environments.

Wricke’s ecovalence (W^2^) was used to compare the behavior of each clone to the average behavior of all clones studied to identify those that interacted with the environment (Wricke, 1962; Kang et al., 1987).

## Results

### Estimation of the effects of genotype, environment, and G×E interaction

**Figure 2:**
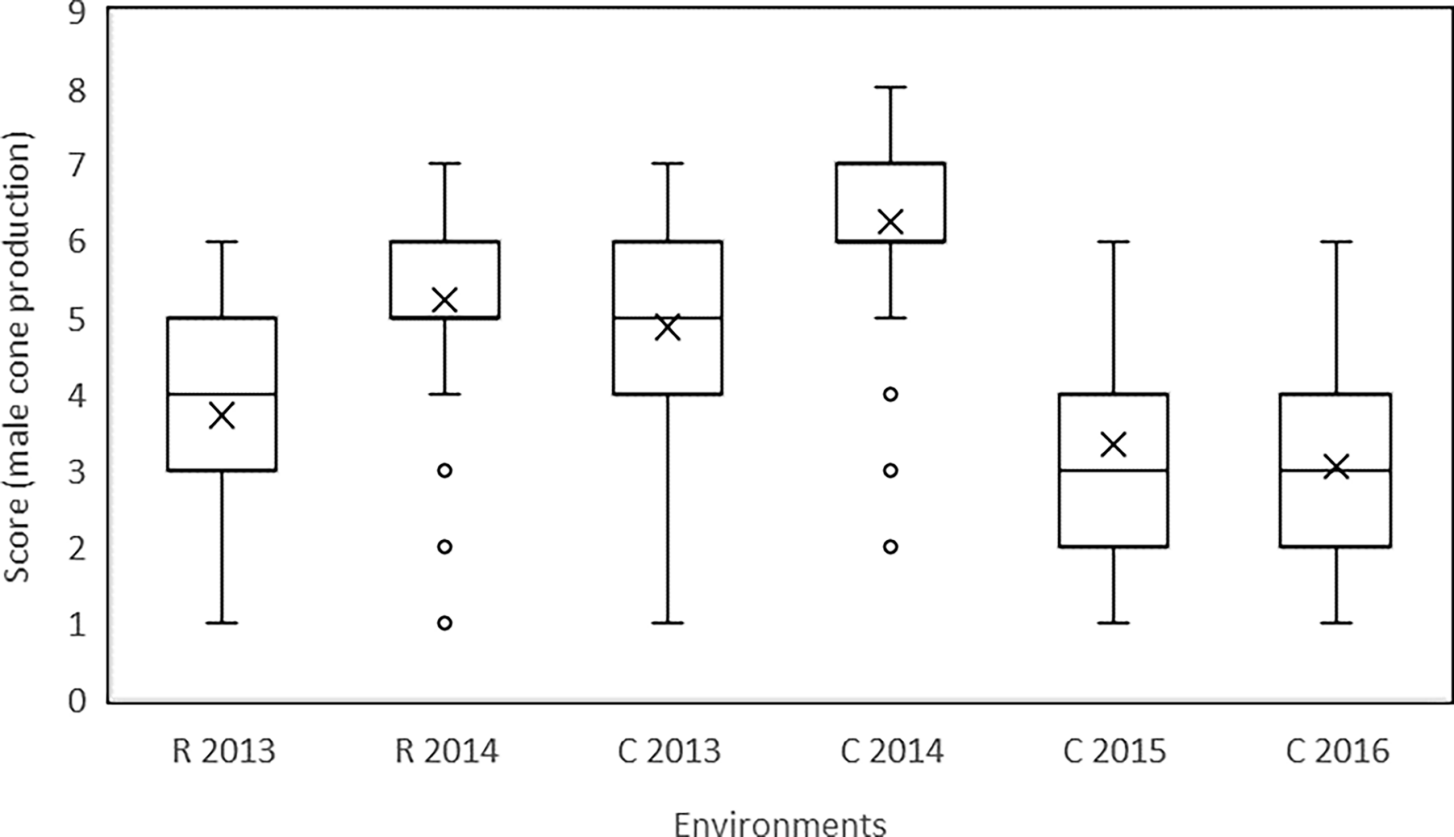
Data referred to the scores recorded for male cone production of individual ramets of the surveyed common cypress clones in the two sites (R: Roselle; C: Cannara) and in various years are reported as box plot. The ends of the box are the first and third quartiles, the line inside the box is the median value (the mean value is also reported as x), the two vertical lines outside the box extend to the highest and lowest observations; data not included between 1.5 IQR (outliers) are plotted with a dot.

The production of male cones varied significantly over the years at both sites (**Table 1**). The highest production of cones both at Roselle (mean score 5.22) and at Cannara (mean score 6.23) was recorded in 2014. In 2013 and 2014, Cannara showed higher scores than Roselle based on the clones replicated at both sites. The variation in the mean score of clones among years in a single site was greater than the variation in the mean score between sites. Also, the range of score variation recorded for the examined clones showed differences between the various years at the two sites. In 2014, the highest clone scores were 7.00 in Roselle and 8.00 in Cannara, and the lowest scores were 2.8 in Roselle and 2.5 in Cannara. In the other years, the clones with the lowest score had a value of 1.0 or 1.3, showing null (or almost null) male cone production (**Table 1**). The boxplots in **Figure 2** show the variation in the scores of the individual ramets recorded in the two sites in the various years. In 2014, 50% of the ramets (those included between the 25th and 75th percentiles) scored between 6.0 and 7.0 in Cannara and between 5.0 and 6.0 in Roselle. In Cannara, in 2015 and 2016, the clones included between the 25th and 75th percentiles scored between 2.0 and 4.0.

**Table 1:** Summary of ANOVA of male cone production of *C. sempervirens* clones.

Single/combined environments	Mean scores	Range of mean scores	Genotype (G)		Environment (site or year) (E)		GxE interaction		Error	
			d.f.	MS	d.f.	MS	d.f	MS	d.f	MS
Roselle										
2013 (se)	3.71	1.33-5.80	152	4.65*					504	0.86
2014 (se)	5.22	2.8-7.0	152	3.92*					504	0.39
2013-2014 (cy)	4.46	2.5-6.3	152	6.11*	1	694.2*	152	2.42*	1008	0.63
Cannara										
2013 (se)	4.91	1.0-7.0	97	6.67*					341	0.41
2014 (se)	6.23	2.5-8.0	97	5.11*					343	0.37
2015 (se)	3.33	1.0-6.0	97	7.50*					342	0.29
2016 (se)	3.05	1.0-6.0	97	10.3*					343	0.56
2013-2016 (cy)	4.38	1.65-6.45	97	18.9*	3	927.8*	291	3.49*	1369	0.41
Roselle-Cannara										
2013 (cs)	4.44	1.0-7.0	98	8.67*	1	134.8*	98	3.65*	580	0.60
2014 (cs)	5.82	2.25-7.40	86	4.38*	1	95.8*	86	2.31*	528	0.34

Data were separately analyzed for each environment (each site x year combination), and for combined environments (distinct years within a site and between the two sites in 2013 and 2014). MS values and degree of freedom are reported for each analysis for the factors included. Asterisk means significance for *p*< 0.001. se, single environment; cy, combined years (within a same site); cs, combined sites

ANOVA for single environments showed in all years of the survey a highly significant effect of genotype on the production of male cones at both sites (**Table 1**). The combined ANOVA showed highly significant effects of genotype, environment (both in the joint analysis of the two sites and in the joint analysis of the years in a single site), and G×E interaction. Environment-related effects accounted for the highest proportion of total variance observed. MS values related to environment were in fact always much higher than the MS values of genotype and G×E interaction (**Table 2**). The MS values of the random error due to intra-clonal variation were relatively small.

**Table 2:** ANOVA variance component analysis of male cone production of *C. sempervirens* clones.

Single/combined environment	Environment (site or year) (F value) fixed	Clone $\;\;\sigma_{c}^{2}$	Genotype x environment $\sigma_{ExC}^{2}$	error $\;\sigma_{e}^{2}$	Phenotypic $\sigma_{P}^{2}$	$R_{b}^{2}$ ± SE	$R_{c}^{2}$ ± SE
Roselle							
2013 (se)		0.87 (50.6%)	–	0.85 (49.4%)	1.09	0.50± 0.01	0.80 ± 0.008
2014 (se)		0.82 (67.8%)	–	0.39 (32.2%)	0.92	0.67 ± 0.04	0.89 ± 0.02
2013-2014 (cy)	1097.17*	0.42 (28.8%)	0.41 (28.1%)	0.63 (43.1%)	0.72	0.29 ± 0.04	0.60 ± 0.03
Cannara							
2013 (se)		1.39 (77.2%)	–	0.41 (22.8%)	1.49	0.77 ± 0.03	0.93 ± 0.01
2014 (se)		1.05 (74.5%)	–	0.36 (25.5%)	1.13	0.74 ± 0.03	0.92 ± 0.01
2015 (se)		1.60 (84.6%)	–	0.29 (15.4%)	1.67	0.84 ± 0.02	0.95 ± 0.007
2016 (se)		2.16 (79.4%)	–	0.56 (20.6%)	2.30	0.79 ± 0.03	0.94 ± 0.009
2013-2016 (cy)	2260.08*	0.86 (44.1%)	0.68 (34.9%)	0.41 (21.0%)	1.08	0.44 ± 0.03	0.81 ± 0.02
Roselle-Cannara							
2013 (cs)	224.9*	0.73 (33.9%)	0.83 (38.6%)	0.59 (27.5%)	1.25	0.33 ± 0.05	0.58 ± 0.04
2014 (cs)	280.9*	0.33 (27.8%)	0.52 (43.7%)	0.34 (28.5%)	0.64	0.27 ± 0.04	0.51 ± 0.04

In the combined environment analysis, genotype (clone) and GxE interaction were considered as random effects, while environment was considered as a fixed effect (the F value was reported). Phenotypic variance, individual tree repeatability ( $R_{b}^{2}$ ), repeatability of clonal means ( $R_{c}^{2}$ ) are also reported. se, single environment; cy, combined years (within a same site); cs, combined sites. Asterisk means significance for p<0.001

### Variance components and clone repeatability

#### Single environment analysis

**Table 2** reports results for the single environment analysis. At Cannara, the component of variance due to clone effect $\left(\sigma_{c}^{2}\right)$ ranged from 74.5% to 84.6% among the 4 years, whereas the variance due to error was contained between 15.4% and 25.5%. At Roselle, the clonal variance component was lower than at Cannara and accounted for 50.6% of the variation in 2013 and 67.8% in 2014 (**Table 3**). Variance due to error was consequently higher than at Cannara in the same 2 years. The repeatability of clonal means $R_{c}^{2}$ ranged from 0.92 to 0.95 at Cannara from 2013 to 2016 and was 0.80 and 0.89 at Roselle in 2013 and 2014, respectively (**Table 3**).

**Table 3:** Genetic intra-trait correlations between mean scores (male cone production) of clones in paired environments.

	C 2013	C 2014	C 2015	C 2016	R 2013	R2014
C 2013	–	–				
C 2014	0.632**	–				
C 2015	0.471**	0.421**				
C 2016	0.421**	0.426**	0.924**			
R 2013	0.542**	0.298*	0.064	0.009		
R 2014	0.356**	0.400**	0.196	0.136	0.486**	

Each environment is a combination between sites and years. C, Cannara; R, Roselle; **p< 0.01 and *p< 0.05.

#### Combined environment analysis

In the analysis of the two sites combined, the significant effects of site and G×E interaction reduced the variance component due to the clone effect. The variance component due to G×E interaction was responsible for 38.6% and 43.7% of the variance observed in 2013 and 2014, respectively, whereas variance due to genotype accounted for 33.9% and 27.8% of the phenotypic variance in 2013 and 2014, respectively (**Table 3**). The clonal means repeatability $R_{c}^{2}$ for the combined sites was 0.58 in 2013 and 0.51 in 2014 (**Table 3**).

The comparison of different years within a single site also highlighted the significant effects of environment and G×E interaction. The 4-year combined analysis showed that at Cannara, the variance component due to clone had a proportionally greater impact (44.1%) than the component due to G×E interaction (34.9%), and $R_{c}^{2}$ was still quite high (0.81; **Table 3**). The 2-year combined analysis showed that at Roselle, the components of variance due to genotype and G×E interaction were proportionally similar (about 28%) and the $R_{c}^{2}\;$ was 0.60 (**Table 3**). The residual error was always greater in Roselle than in Cannara, both in the single and in the combined year analyses.

The intra-trait genetic correlations of clonal means assessed in pairs of environments were mostly significant and were moderate to high, ranging from 0.40 to 0.92 (**Table 4**). The latter value was found in Cannara between scores measured in 2015 and 2016, denoting a consistent response of clones across the 2 years. Nonsignificant correlations were found only when clonal means were paired between different sites and years.

**Table 4:** Mean number and standard deviation of the number of pollen grains per cone counted in high and low producing *C. sempervirens* clones.

High producing clones			Low producing clones		
Clone	grains/ml (mean ± dev.st)	Length x width (mm)	Clone	grains/ml (mean ± dev.st)	Length x width (mm)
3150	183,889 ± 43,931	4.26 x 1.60	3097	303,333 ± 61,018	3.85 x 1.70
3142	273,148 ± 295,142	3.72 x 1.74	3087	256,852 ± 114,781	4.16 x 1.66
3126	261,111 ± 205,048	3.90 x 1.80	3180	27,778 ± 70,437	4.03 x 1.68
Mean	239,383 ± 185.823	3.96 x 1.71	Mean	262,654 ± 80,957	4.01 x 1.68

Mean size of male cones of the sampled clones was also reported. No significant differences were found between the mean number of pollen grains per male cone counted in high cone and low cone producing clones (ANOVA: F= 0.11; *p* > 0.05).

### Characterization of clones and their stability

Regarding the complete list of results for the single clones, the scores recorded for male cone production in the six environments (meant as site × year combinations) are listed in **Supplementary Table 2**.

**Figure 3:**
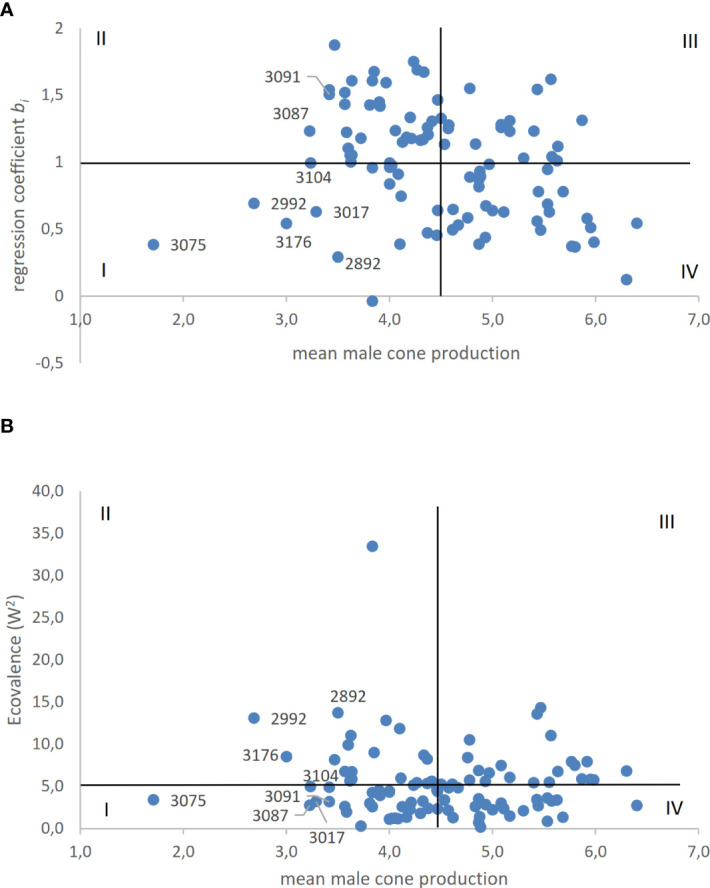
**(A, B)**. The clonal stability regression coefficient **(A)** and Wricke’s ecovalence **(B)** of male cone production of *C. sempervirens* clones are plotted against mean scores of the same clone. Horizontal line represents the mean of the two stability coefficients; vertical line represents the mean score of all clones.

The stability of male cone production among clones was evaluated across the six environments. The two stability parameters calculated for the male cone production of each clone were plotted against the mean score of clones recorded in the six environments (**Figure 3**).

As regards the linear regression coefficient, 48 out of 98 clones showed *b_i_* < 1. These included the clones with the highest and lowest mean scores. With regard to ecovalence, 42 out of 98 clones had a value higher than the average W^2^ value and mostly contributed to G×E interaction. As shown in **Figure 3**, of clones with the lowest production of male cones, only clones 3075, 3017, and 3104 were positioned in quadrant I (low male cone producing and stable clones) based on both stability parameters. Clones 2992 and 3176 (mean scores 2.68 and 3.0, respectively) were positioned in quadrant I based on the regression coefficient *b_i_*, whereas they were positioned in quadrant II (low producing but not stable clones) based on W^2^. Overall, 31 and 18 out of 98 clones were positioned in quadrant I based on W^2^ and *b_i_*, respectively.

In the single environments, various clones had a score less than or equal to 2.0 or even 1.0, which indicates very low or null cone production. At Roselle, 12 clones (out of 151) scored less than or equal to 2.0 in 2013, whereas in 2014 the lowest score recorded for clones was 2.8 (**Supplementary Table 2**). At Cannara, only 3 clones (out of 98) had a score less than or equal to 2.0 (clones 2992, 2892, and 3075) in 2013; only clone 3075 had a score less than 3.0 in 2014. In 2015 and 2016, 20 and 32 clones, respectively, scored less than or equal to 2.0 (**Supplementary Table 2**).

**Figure 4:**
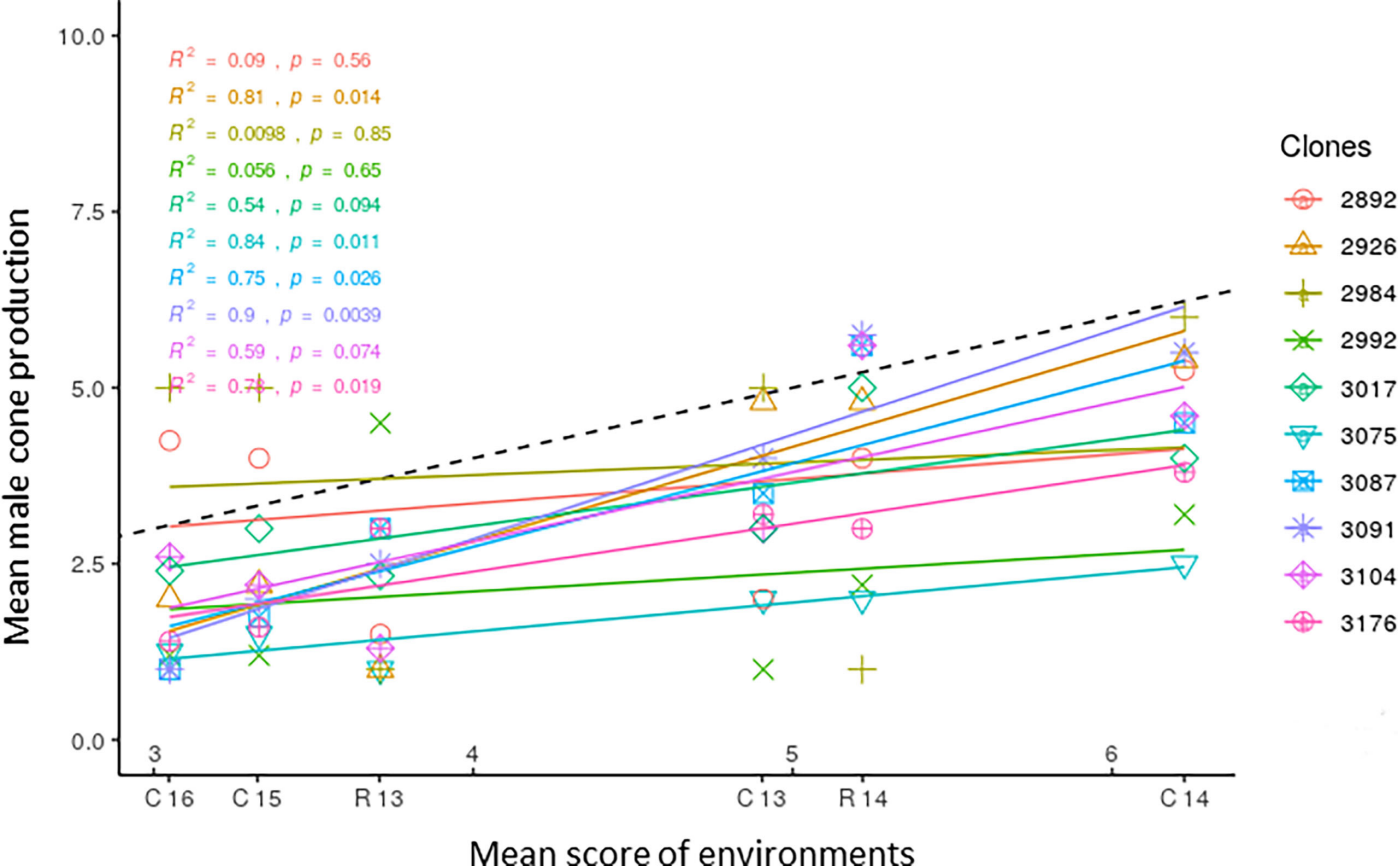
Clonal means of the ten *C. sempervirens* clones showing the lowest production of male cones are plotted against mean scores of the six environments (C13, C14, C15, C16, R13, R14), reporting the corresponding regression lines. R^2^ values and significance level are reported for all regressions. C13, Cannara 2013; C14, Cannara 2014; C15, Cannara 2015; C16, Cannara 2016; R13, Roselle 2013; R14, Roselle 2014.

Mean scores of the nine clones with the overall lowest production of male cones were plotted against the means of each environment, and the obtained regression lines are reported in **Figure 4**. The population mean is also reported; as it was obtained by plotting the score of the single clones against the mean of all clones in each environment, its regression coefficient *b_i_* was 1.0. The plot depicts the different responses of clones to the range of environments. The regression lines of clones 2992, 2984, and 2892 were not significant. Clone 3104 was characterized by a regression coefficient close to 1.0, showing average stability over all environments. Clones 3075, 3017, and 3176 with a regression coefficient *b_i_* < 1 showed good (greater than average) stability, exhibiting little variation in male cone production across environments. Among them, clone 3075 showed the lowest mean score. The other clones (3091, 2926, 3087) had a regression coefficient *b_i_* > 1 and were responsive to high cone producing environments. Of these nine clones, only 3075, 3017, and 3104 also had a W^2^ value below average. The difference between the score of the most stable clones with the lowest production of male cones and the mean score of the clone population was about 2.0 in the environment less conducive to flowering (Cannara 2016) and ranged from 3.0 to 4.0 in the environment more favorable to flowering (Cannara 2014).

### Count of pollen grains per male cone

The number of pollen grains contained within the male cones was rather variable between the examined clones and even within single clones, as shown by the high standard deviation (**Table 4**). However, the average numbers of pollen grains of clones classified as high (239,883) and low (262,654) male cone producing were not significantly different (ANOVA, F = 0.11; *p* = 0.73).

## Discussion

### Clonal variation and repeatability

The present study evaluated for the first time the production of male cones in 20-year-old *C. sempervirens* clones cultivated in two sites in central Italy and studied the variation over same clones at different sites and over the years within sites.

Large differences were found in the production of male cones among clones at both locations. A clear distinction between clones emerged at both sites for the production of male cones, which resulted under important genetic control. The repeatability of clone means $R_{c}^{2}$ was more than 0.90 at Cannara and more than 0.80 at Roselle. Given the influence of the microenvironment on flowering, arrangement in groups along a single row of ramets of a single clone may have reduced the component of variance due to error, thus increasing the repeatability of clonal means $(R_{c}^{2}$ ) compared to a random distribution of ramets.

These findings mean that the selection of *C. sempervirens* clones based on the number of male cones produced in a given environment is feasible. Moreover, this study relied on clones reasonably capable of fully expressing flower production based on their genetic potential, as in both sites the ramets were 20 years old at the beginning of the surveys, and common cypress starts producing male cones already at the age of 4–5 years (Farjon, 2005).

However, the production of male cones showed significant variation between the two sites, both in 2013 and 2014 and between years within a single site.

In regard to the single environment analysis (same site and year), in the combined analysis the proportion of variance due to genotype was significantly reduced as a result of the significant effect of G×E interaction. The $\sigma_{ExC}^{2}$ variance component accounted for a proportion greater than or equal to $\sigma_{c}^{2}$ , except in the combined analysis between years in Cannara. At this site, $R_{c}^{2}$ was still quite high, which highlights the importance of the genotypic variance component in the total variance of clones in consecutive years. In the 2-year combined analysis conducted in Roselle, $R_{c}^{2}\;$ was lower than at Cannara because of the higher error variance component for the former site. In the combined analysis between sites, $R_{c}^{2}$ and $R_{b}^{2}\;$ were even lower than in the combined analysis for years at a single site because of the higher proportion of the $\sigma_{ExC}^{2}$ variance component. This suggests that selection for male cone production may be more difficult across sites than across years in a single site, despite the fact that the greatest variations between mean scores of clones were reported between different years in a single site.

However, the estimation of the variance component due to G×E interaction is influenced by the fact that in the variance component analysis, environment is considered a fixed or random effect (Burdon, 1977). The estimation of genetic correlations between environments is not affected by this and provides a direct indication of the role of the environment in generating interaction (Burdon, 1977). In the face of a significant G×E interaction, in this study the intra-trait genetic correlations (type B) of clonal mean scores between pairs of years in a single site and between different sites in a single year were positive and significant. This indicates that variation in male cone production was fairly consistent across years and across sites in the population of clones examined.

The between-year variation was clear at both sites, as indicated by the high MS values. In particular, 2014 was the year in which the highest production of male cones was recorded at both sites. The favorable combination of precipitation and temperature recorded at both sites starting in the summer of 2013 may have led to the particularly abundant flowering in 2014. Based on the recorded climate data, the highest production of male flowers recorded in 2014 could be due to the abundance of rain in both 2013 and 2014 (more than 1000 mm) and the definitely mild temperatures registered in January–March 2014 at both sites. These conditions may have promoted flower initiation and bud differentiation in the summer of 2013 (which was particularly rainy) and the subsequent development of cones up to full flowering in the winter of 2014. Some authors in fact have reported a positive effect of watering during the growing season on the production of pollen cones in pine species (Puritch, 1977; Li et al., 2021). The climatic conditions in 2014 could represent a sort of stress test for assaying the production of male cones by common cypress clones in a particularly conducive environment. The absence of frost events, which generally cause the premature fall of unripe male cones during the winter, may have favored their development toward the full flowering phase. The data collected in 2014 are therefore useful for evaluating the production of male cones by clones in a particularly conducive environment.

Cannara was a more favorable site for flowering than Roselle, both in 2013 and in 2014. In Cannara the more clayey soil and the higher and more regular rainfall allowed for a greater availability of water than in Roselle, which was characterized by sandy loam soil and lower rainfall. This promoted greater growth of plants in Cannara and may have favored the production of cones. Although correlations between clone dimensions and flowering abundance were not investigated, the differences in male cone production observed between the two sites in 2013 and 2014 could have been due to the better growing conditions plants experienced in Cannara compared to Roselle, as reported in the study by Nocetti et al. (2015) on *C. sempervirens* clones selected for timber production.

The fluctuations in the production of cones observed over the years, as well as the direct effect of weather conditions, may have been due to the alternation of flowering and fruiting according to natural cycles of two or more years, as observed in many forest tree species, and 2014 may have been a mast year. In this regard, one of the recognized benefits of this large flowering effort is successful wind pollination (Smith et al., 1990).

However, the average production of male cones in the six environments shown in **Figure 4** illustrates the importance of seasonal fluctuations (from one year to the next) as a major factor of environmental variability. The greatest difference between the mean scores of the environments was in fact found in Cannara between 2016 and 2014. Therefore, the differences in soil and climate between the two sites seem to affect the environment relatively less compared to seasonal climatic fluctuations in relation to the production of male cones by cypress clones. The average production of male cones of a wide range of genotypes represents a synthetic measure of the value of the environment in relation to this parameter (Finlay and Wilkinson, 1963). This proxy provides a useful method of describing the complex of factors determining a natural environment and classifying environments as combinations of sites and seasons.

Various studies have evaluated annual variation in flower production in clonal orchards of different conifer species with the aim of estimating genetic variation in the seed crop, and all have shown significant differences between clones and years (Saito and Kawasaki, 1984; Burczyk and Chalupka, 1997; Tsubomura et al., 2012). Nikkanen and Ruotsalainen (2000) reported large fluctuations between years in 67 *Picea abies* clones, with a single tree repeatability ( $R_{b}^{2}$ ) of male flower production ranging from 0.30 to 0.49. Similar results were obtained in a study by Kang (2000) on 99 *Pinus densiflora* clones conducted over four consecutive years, which found a repeatability for single trees between 0.19 and 0.27. Bilir et al. (2006), analyzing 25 clones of *P. sylvestris* in three different orchards, found rather low single tree repeatability values ranging from 0.16 to 0.23. Low to moderate $R_{b}^{2}$ values were found for male cone production in two clonal banks of *P. koraiensis* (Choi et al., 2004). In all these studies, the single tree repeatability was lower than the value found in our study of common cypress.

### Pollen grains contained in male cones

The number of pollen grains contained in the cones soon before their opening did not differ significantly between clones classified as high and low cone producing. Furthermore, the amount of pollen produced and released by a *C. sempervirens* tree is fundamentally related to the number of male cones that reach maturity and the opening phase. This confirms the validity of the selection of low pollen emitting clones based on the reduced production of male cones. A similar number of pollen grains per cone (range 333,433–383,600) was found by Hidalgo et al. (1999) in a comparison of *C. sempervirens*, *C. macrocarpa*, and *C. arizonica*. Likewise, no significant differences in the number of pollen grains per cone were found between adult *C. sempervirens* trees by Aboulaich et al. (2008) in Morocco (mean 460,778; range 386,000–504,667). Compared to these two works, the number of pollen grains per cone found in the *C. sempervirens* clones examined in the present work was lower. This discrepancy may be due to various factors—different counting methods, the development of the cones when harvested, the size of the cones at flowering (cones in the present study were slightly smaller than those measured by Aboulaich et al., 2008)—which are in turn linked to environmental conditions.

### Clonal stability and selection of low male cone producing clones

In this study, the stability of the clones across the six environments represented by each site per year combination was evaluated based on the dynamic concept, which assumes that organisms are responsive to environmental changes. The linear regression coefficient allowed us to evaluate stability and hence relative adaptability (the ability to respond well to a wide range of environments). With ecovalence (W^2^), a stable genotype shows a yield response in each environment that is always parallel to the mean response of all genotypes, and thus clones that interact with the environment can be identified (Annicchiarico, 2002). The plots obtained by associating the stability parameters with the average scores of clones are useful for selection (**Figure 3**). About 50% (48 out of 98) of the clones showed a regression coefficient *b_i_* < 1 and production of male cones across environments that varied to a lesser extent than the average. With regard to ecovalence, 41 out of 98 clones had a W^2^ value lower than the average, showing a smaller effect on G×E interaction of the population of clones. Therefore, clones characterized by stability were quite common in the examined population. Clones positioned in quadrant I (bottom left) are those to be preferred for the lowest score and greater stability. The main goal is to identify stable clones among those with scores lower than the 15th percentile (3.61). In plots obtained with both the regression coefficient *b_i_* and W^2^, the most suitable clone was 3075, which had the lowest mean score of all clones tested in the six environments. Only two other clones (3104 and 3017) had reduced cone production (below the 15th percentile) and were stable based on both parameters.

In the plot in **Figure 4**, the slopes of the regression lines of the 10 clones with the lowest average score depict their different behavior across environments. Only clones 3075, 3176, and 3017, among those with a significant regression coefficient, had *b_i_* < 1, showing little change in cone production across environments and greater stability. They were less sensitive to environmental change and had scores lower than average in the low cone producing environments and much lower than average in the more favorable environments. Three clones (2992, 2984, and 2992), apparently stable (*b_i_* < 1), had non significant regression coefficients. However, some unstable clones with *b_i_* > 1 (3091, 2926, 3087) were responsive to the most favorable environments and increased their scores to a greater extent than the average of the clone population, showing phenotypic plasticity. Clone 3104 with *b_i_* = 0.99 showed a type of adaptation (i.e., the ability to perform well in different environments), producing fewer male cones than average. Adaptability is commonly used in studies on the yield performance of crops and tree growth, which are more directly related to soil and climate conditions (Finlay and Wilkinson, 1963; Yu and Pulkkinen, 2003; Nelson et al., 2018). In this study adaptability was less exploited, because the effect of environmental conditions on flowering is less direct and because selection in this study was oriented toward clones with a lower yield (in terms of cones produced) and stability.

The cypress clones with the highest production of male cones showed little variation in their score across environments, and 8 out of the top 10 in the ranking showed *b_i_* < 1 (from 0.12 to 0.77). They were not very sensitive to environmental change and maintained a high production of cones, showing low plasticity (**Supplementary Table 2**).

The ability to maintain reduced cone production in all environments is a priority in the selection of cypress genotypes with low allergenic potential. The clones that show low production in less conducive sites, but also show plasticity (i.e., they rapidly increase production in favorable environments), are not very reliable for exploitation in green areas in a wide range of environments in urban and peri-urban settings.

The findings of the present study suggest that among *C. sempervirens* it is possible to select clones genetically characterized by low production of male cones and therefore of pollen. However, breeding for stable clones characterized by constant null production of male cones across environments appears to be a difficult task. Some clones (such as 3075, 2992, 3087, 3091, 2926, 2984) showed null production of male cones in some environments, but all were more or less subjected to variation across years or sites. Among these, 3075, with the lowest male cone production, was also stable, showing low sensitivity to high cone producing environments, and was the best clone. Other clones (3104, 3017, 3176), although they never showed null cone production, had very low scores and were also stable. The low cone producing clones identified in this study allowed for a reduction in the production of male cones by up to 10 times compared to the average in the same environment.

In Japan, a genetic improvement activity aimed at obtaining seedlings with reduced production of male cones has been carried out on *C. japonica* to address the problem of allergy to the pollen of this species in the population. In a study conducted by Tsubomura et al. (2012), an estimated –50% of genetic gain in male flower production in progeny was considered by the authors an enormous achievement. The present work, which was based on an assessment of clonal material, allows for a greater gain than a design based on the inheritance of male flower production.

To select male-sterile clones characterized by null pollen production, it might be useful to study the behavior of full-sib progenies derived from the crossing of hypo-producing pollen genotypes, such as those identified in this study, although segregation may lead to the loss of other valuable traits in offspring. Eventually male sterility could be induced in selected genotypes through the regulation of genes involved in the development of the pollen grains (Höfig et al., 2006; Zhang et al., 2012).

According to the World Health Organization, and following the VII European Environment Action Program, many cities are reinforcing and implementing green infrastructure as essential measures in strengthening their resilience to climate change and mitigating its effects, improving air quality, and reducing pollution. In the creation of green spaces, priority is given to tree species based on their effectiveness at reducing particulate matter (PM) pollution, according to specific rankings. *C. sempervirens* ranked at the top among 100 species evaluated on their PM removal efficiency based on the density and fine texture of the canopy, the reduced size of the leaves, and their rough and scaly surface (Yang et al., 2015). However, cypress is known for the impact of allergenic pollen emissions intrinsic to the species, and among 150 ornamental urban trees and shrubs it was considered to have a very high Value of Potential Allergenicity (Cariñanos and Marinangeli, 2021). In this context, the clones of *C. sempervirens* evaluated as low pollen producers in the present work have value related to both their ability to control PM pollution and a reduced allergenic potential. The low male cone producing clones identified can be useful candidates for creating public green areas, especially in urban settings, reducing the load of allergens on the allergic population and limiting the risk of sensitization in non-allergic subjects, also mitigating proximity pollinosis (Seitz and Escobedo, 2009). They also may meet guidelines for the use of low pollen producing plants in the design and planning of urban green spaces with low allergy impact (Cariñanos and Casares-Porcel, 2011).

## Conclusions

In regards to previous studies on cypress pollen production, evaluation of the genetic parameters of male cone production in *C. sempervirens* was accomplished herein. The goal was to investigate the possibility of selecting genotypes with a reduced allergenic potential to respond to the increasing prevalence of cypress pollen allergies in the Mediterranean population (D’Amato et al., 2014; Charpin et al., 2019).

Despite an important genetic control of male flowering, the incidence of environmental conditions and GxE interaction on the variability of pollen production in clones of *C. sempervirens* emerged. Male flowering was subject to fluctuations over the years and across sites, and even clones that showed null production of male flowers in a given environment underwent some variation over time or site. The low male cone producing clones identified showed a reduction in male cone production from 15 to 25 times compared to clones with higher production and from 5 to 10 times the mean production recorded in the same environment. These results represent a fundamental step in studying variation in pollen production in *C. sempervirens* and selecting clones characterized by reduced production of male cones to address pollen allergies. These clones are promising resources for reducing atmospheric pollen in green spaces in urban settings. The selection of male-sterile clones with valuable aesthetic traits appears to be difficult to achieve with classic breeding programs but can be pursued through the adoption of gene editing techniques.

## Data availability statement

The original contributions presented in the study are included in the article/**Supplementary Material**. Further inquiries can be directed to the corresponding author.

## Author contributions

RD conceived the idea and designed the experiments. RD, SB, VL, and GR visually inspected trees and collected samples for flower and pollen counts. RD analyzed the data and performed the genetic analysis. SB and GR performed the microscopic analysis. RD, SB, and GR wrote the manuscript. RD was financially responsible for the project. All authors contributed to the article and approved the submitted version.

## Funding

This work was supported by the CNR and Regione Toscana project CYPALL (Indagine sulle relazioni cipresso-allergie: selezione di varietà di cipresso con polline a ridotta allergenicità e con indotta sterilità).

## Acknowledgments

The authors wish to thank Dr. Paolo Raddi for his valuable help with data collection and for critically reading the manuscript and Dr. Edoardo Scali for his help plotting the regression lines for stability in R.

## Conflict of interest

The authors declare that the research was conducted in the absence of any commercial or financial relationships that could be construed as a potential conflict of interest.

## Publisher’s note

All claims expressed in this article are solely those of the authors and do not necessarily represent those of their affiliated organizations, or those of the publisher, the editors and the reviewers. Any product that may be evaluated in this article, or claim that may be made by its manufacturer, is not guaranteed or endorsed by the publisher.
